# Efficacy of Ahmed Glaucoma Valve Implantation on Neovascular Glaucoma

**DOI:** 10.7150/ijms.35267

**Published:** 2019-09-20

**Authors:** Zhan Xie, Hai Liu, Mulong Du, Min Zhu, Sean Tighe, Xue Chen, Zhilan Yuan, Hong Sun

**Affiliations:** 1 Department of Ophthalmology, Jiangsu Province Hospital, Nanjing, Jiangsu Province 210029, China; 2 Department of Ophthalmology, The Second People's Hospital of Yunnan Province (Fourth Affiliated Hospital of Kunming Medical University); Yunnan Eye Institute; Key Laboratory of Yunnan Province for the Prevention and Treatment of ophthalmology (2017DG008); Provincial Innovation Team for Cataract and Ocular Fundus Disease (2017HC010); Expert Workstation of Yao Ke (2017IC064), Kunming 650021, China; 3 Department of Biostatistics, School of Public Health, Nanjing Medical University, Nanjing, Jiangsu Province 210029, China; 4 Public Health, the University of Arizona, Tucson, Arizona, 85709, USA; 5 Tissue Tech, Inc., Ocular Surface Center, and Ocular Surface Research & Education Foundation, Miami, FL 33173, USA

**Keywords:** Ahmed glaucoma valve, influence factor, neovascular glaucoma, surgical success rate

## Abstract

To evaluate the efficacy of Ahmed glaucoma valve (AGV) implantation in treating neovascular glaucoma (NVG) and analyze the factors influencing the surgical success rate, a retrospective investigation of 59 NVG patients (66 eyes) who underwent AGV implantation was conducted at Jiangsu Province Hospital, China, from January 2014 to June 2018. Intraocular pressure (IOP), visual acuity, surgical success rates, medications, and complications were monitored at post-operative 1 day, 1 week, 1, 3, 6 and 12 months. Surgical success criteria were defined as 6 mm Hg < IOP < 21 mmHg with or without additional medications. Results showed average IOP was statistically significant between pre-operative visit and each follow-up visit (all P<0.05). At 12 months, the success rate was 66.7%. Multiple stepwise regression analysis suggested that age, panretinal photocoagulation (PRP), complications and hyphema were significant factors influencing the surgical success rate (all P<0.05). Thus, we conclude that AGV implantation is effective and safe for treatment of NVG. Surgical success is dependent on age, PRP, complications, and hyphema.

## Introduction

Glaucoma is a leading cause of irreversible blindness globally, which may affect up to 111 million people worldwide by 2040 1-3. Neovascular glaucoma (NVG), a severe secondary glaucoma, is closely related to retinal ischemic diseases. Ischemia triggers the release of various angiogenic factors, including vascular endothelial growth factor (VEGF), penetrating the anterior chamber to cause neovascularization of the iris and angle. Recently, with the increasing incidence of diabetes and vascular diseases, NVG is also increasing steadily, accounting for more than 30% of refractory glaucoma 4. Therefore, selection of optimal treatments for NVG has become the focus to many ophthalmologists in clinics worldwide. Unfortunately, NVG patients usually respond poorly to anti-glaucoma drugs. Therefore, appropriate surgical interventions are usually required, including ciliary body destructive surgery, drainage valve implantation, and trabeculectomy with mitomycin C. Of these treatment options, drainage valve implantation has become increasingly popular for treatment of NVG patients due to the potential for severe complications in ciliary body destructive surgery and low success rates observed in conventional trabeculectomy surgery 5, 6.

Since 1993 when Ahmed glaucoma valves were first introduced in clinical practice, eye specialists around the world have gradually recognized their potential in the treatment of NVG 7, 8. The Ahmed valve has a one-way pressure-sensitive control valve, which restricts the drainage device to be operative only under an IOP of 8 to 14 mmHg, thus preventing early and late surgical complications, including excessive aqueous drainage, shallow anterior chamber and intraocular hypotension. Previously, Ahmed glaucoma valve (AGV) implantation was reserved for glaucoma patients poorly controlled after one or more filtration procedures, however recent evidence has recently encouraged its use as a primary surgery in refractory glaucoma such as secondary to neovascular glaucoma (NVG), pars plana vitrectomy (PPV), penetrating keratoplasty, and uveitis 9. Unfortunately, the AGV has limitations of a small surface area and hypertensive phase 10-13 and its reported success rate varies greatly depending on the follow-up period and types of glaucoma 10, 14-16. Importantly, effect of AGV on treatment of specific NVG has received little attention from the scientific world so far. Therefore, we have designed this study to evaluate the efficacy of AGV in treating NVG. Herein, we report the results of a retrospective study on 59 NVG patients (66 eyes) that underwent Ahmed glaucoma valve (AGV) implantation from January 2014 to June 2018. Such results may provide the scientific guidelines for clinicians to treat this disease effectively and successfully.

## Materials and Methods

**Data Sources** We retrospectively reviewed the medical records of patients with NVG who underwent AGV implantation (model: FP7, New World Medical Inc., Rancho Cucamonga, CA, USA) at Jiangsu Province Hospital, China, between January 2014 and June 2018. This study was performed according to the Declaration of Helsinki and its subsequent revisions, and the ethics approval was obtained from Jiangsu Province Hospital Medical Ethics Committee.

**Inclusion Criteria** NVG patients were diagnosed clinically. NVG diagnostic criteria included typical iris neovascularization and ectropion uveae of pupillary margin, trabecular meshwork neovascularization, peripheral anterior synechia, increased IOP, decreased visual acuity, characteristic visual field defect, characteristic glaucomatous cup, and previous primary disease. Other inclusion criteria included: IOP maintained higher than 21 mm Hg after applying IOP-lowering medications, post-operative follow-up period >12 months, and patient without severe systemic or mental diseases except primary glaucoma disease.

**Exclusion criteria.** Patients were excluded if they were <14 years old, had less than 12-month follow-up, or were in poor general physical conditions (such as unsatisfactory blood glucose control in diabetics).

**Sample size:** A total of 90 candidate patients were initially enrolled in this study. After review of inclusion and exclusion criteria, 66 patients (73.3%) were enrolled for further analysis. Their basic information was summarized in Table 1.

**Surgical Method** All surgeries were completed by the same experienced glaucoma specialist. The surgical procedure was as follows:(1) 2.5 ml of 2%lidocaine + 0.75% bupivacaine (mixed at a ratio of 1:1) was applied for peribulbar anesthesia. (2) With a suspension wire made in the corneal limbus, the bulbar conjunctiva above the temple was cut along the corneal limbus, and a fornix-based conjunctival flab was created to expose 2 recti muscles. A pocket was performed between the episclera and tenon's capsule by blunt dissection. After the fascia was separated, hemostasis was drained by cautery. A 4x4 mm sclera flap with a 50% scleral thickness was made above the temple. (3) The tube of the valve was irrigated with balanced saline solution to open the valve mechanism. Then the Ahmed valve was inserted between the lateral rectus and superior rectus, and a 6-0 suture was fastened at 10 mm behind the corneal limbus. (4) A lateral corneal incision was made, sodium hyaluronate was injected into the anterior chamber, and an anterior chamber puncture was made in the corneal limbus under the sclera flap. The drainage tube was inserted into the anterior chamber from the puncture site. (5) The sclera flap was closed with 10-0 suture, the drainage tube was fixed and partly ligated for 1 stitch with 8-0 absorbable suture, and the conjunctival flap was sutured.

**Evaluation criteria of surgical efficacy** NVG patients were usually accompanied by diseases of the ocular fundus, such as diabetic retinopathy (DM) and central retinal vein occlusion (CRVO), which have led to poor visual performance and affected examinations of the visual acuity, visual field and OCT. Therefore, visual acuity, cup-disc ratio, visual field, and retinal nerve fiber layer thickness may not be treated as the criteria of surgical success. In this research, IOP served as the major indicator for evaluating surgical success or failure.

**Evaluation of Surgical Success or Failure** (1) Success: IOP was 6 to 21 mmHg without antiglaucoma medications post-operatively. (2) Failure: IOP was lower than 6 mmHg or higher than 21 mmHg after applying antiglaucoma medications post-operatively. In such cases, severe eye complications were observed, for example, retinal detachment and endophthalmitis.

**Follow-up Period** Regular follows-ups were conducted at 1 day, 1 week, 1, 3, 6, and 12 months after surgery.

**Observational Indexes** Best corrected visual acuity (BCVA) was tested by an optometrist using a standard logarithmic visual acuity chart (Yuehua Medical Apparatus and Instruments, Inc., Shantou, Guangdong, China) at 5 meters. The observational indexes included best corrected visual acuity (BCVA, Standard Logarithmic Visual Acuity E Chart), preoperative and post-operative IOP (Goldmann applanation Tonometer, Hagg-Streit, Switzerland), slit-lamp (BM900, Switzerland) microscopic examination, type and number of local and systemic applications of antiglaucoma medications and postoperative complications.

**Statistical analysis** We have enrolled all available patients for analysis and compared multiple levels about clinical efficacy, including the IOP in pre vs. pro-surgery, and stepwise regression analysis for surgical success rate at 1-year follow-up, depending on the research purposes. All statistical analyses were conducted using the SPSS 21.0. For continuous variables, t-test or paired t-test was performed for data with normal distribution, while a corresponding non-parametric test was used for abnormal distribution data. For categorical variables, Pearson's chi-square test or Fisher's exact test was used. A multiple stepwise regression analysis was used for the effect evaluation of the candidate risk factors. A p-value of less than 0.05 was considered statistically significant.

## Results

**Figure 1:**
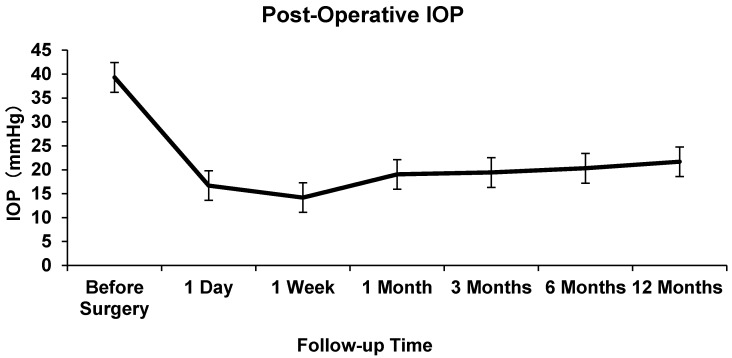
Intraocular Pressure at Baseline and Follow-up

The mean pre-operative IOP was 48.23±8.17 mmHg. At 1 day, 7 days, and 1,3, 6, and 12 months after AGV implantation, the mean IOP was 16.70±10.79, 14.19±6.03, 19.03±8.00, 19.43±5.59, 20.31±5.96 and 21.68±6.64 mmHg respectively. The difference between the mean baseline IOP and the IOP at each follow-up point was statistically significant (P<0.05, Figure 1).

A significant decrease in BCVA at 1 year post-operatively was noted (P < 0.05) compared with the baseline BCVA. The success rate in the entire study population was 66.7% at 1 year after the operation. An average of 2.58±0.56 antiglaucoma medications were applied in all patients pre-operatively, while an average of 1.03±1.05 medications was applied post-operatively at the last follow-up, which was statistically significant (P<0.001).

The most common post-operative complication was hyphema, which could be seen in 10 eyes (15.2%). Nine eyes developed shallow anterior chamber (13.6%). Two eyes had malignant glaucoma (3%). Other post-operative complications included corneal endothelial decompensation (1.5%), choroidal detachment (1.5%), drainage valve exposure (1.5%), low tension retinopathy (1.5%) and drainage valve displacement (1.5%) (Table 2).

Single factor analysis for failure was performed. Factors such as age, gender, course of disease, PRP history, primary disease, lens status, pre-operative anti-VEGF therapy, pre-operative IOP, pre-operative BCVA, pre-medications number, previous operation history and surgical complications were included. A multiple stepwise regression analysis ascertained the relative predictive ability of these factors. Our results suggest that age, PRP, no post-operative complications and post-operative hyphema are statistically-significant factors influencing the surgical success rate (Table 3).

## Discussion

Previously, Yalvac et al reported the 1-year surgical success rate of 38 NVG eyes receiving Ahmed valve implantation was 63.3% 17 and Netland reported a 1-year surgical success rate of 73.1% in 38 eyes 18. Similarly, our results showed a success rate of 66.7% at 12 months in Asian patients.

Risk factors for effectiveness of glaucoma valve implantation are still unclear. Sidoti et al performed Baerveldt drainage valve implantation in 36 NVG patients and discovered that being young was a risk factor of surgical failure 19. Tsai et al 20 and Takihara et al 6 performed filtering surgery on NVG patients with a similar conclusion. Mermoud et al discovered in their research that NVG patients aged over 55 years had a higher Molteno surgical success rate than those aged below 55 years 21. Based on these data and our current results, age is an important factor influencing surgical success rate. This may be linked to the stronger wound healing response in younger patients, making them more likely to develop fiber-wrap in the periphery of the drainage disc, as well as more aggressive illness when the younger patients develop NVG. Therefore, we suggest that clinicians should strengthen the disease education for young NVG patients, make pre-operative disease evaluation, and optimize the treatment plan and follow up after treatment.

Hamard et al believed that for NVG, doctors should not only treat the primary disease, but also improve the pathological status of retinal ischemia 22. One potential way is PRP, which ablates the ischemic retina to decrease tissue oxygen demand, thus reducing VEGF release and the formation of NV. Evans et al found that neo-vessels of iris disappeared in 68% of NVG patients after sufficient retinal photocoagulation. In this study, we found that retinal photocoagulation history was a factor influencing the success rate of surgery 1 year after AGV in NVG patients (P<0.01). Patients who did not receive retinal photocoagulation therapy were more likely to fail after the implantation of Ahmed valve. Therefore, we believe that clinicians should timely conduct standardized retinal photocoagulation after implantation of Ahmed valve drainage for NVG patients. Based on the treatment of primary diseases leading to NVG, standardized retinal photocoagulation is the fundamental method to promote retinal angiogenesis regression and inhibit the growth of retinal angiogenesis.

Another potential risk factor is hyphema. In the research by Choo J et al, postoperative hyphema could be associated with increased conjunctival inflammation and scarring, leading to a higher risk of failure 23. Shunji Nakatake et al discovered in their research that post-operative hyphema increased concentrations of some cytokines may lead to a failure of conjunctival bleb formation 24. Our research confirmed incidence of post-operative hyphema is a risk factor for failure, resembling what has previously reported.

Recently, the intravitreal injection of anti-VEGF drugs has shown promising results in regression of neovascularization 25-27. Adjuvant anti-VEGF treatment may lead to regression of NV in the iris and angle, thus, reducing the incidence of hyphema, providing time and conditions for the subsequent panretinal photocoagulation, therefore, potentially enhancing the surgical outcome of AGV implantation in NVG 6, 28. However the effect of anti-VEGF drug may be transient, hence repeated injections are sometimes required. Additionally, Kwon et al demonstrated pre-operative injection of anti-VEGF drugs increases the success rate of AGV implantation when peripheral anterior synechiae is less than ½ 29. Our study concluded similarly that preoperative anti-VEGF therapy was not associated with a better surgical success at 1 year after drainage valve implantation. This may be due to regression of iris neovascularization which may persist for 8-10 weeks after intraocular injection but then return to its previous condition within 6 months after administration, thus playing a limited, temporary role in the treatment of NVG. Most patients in this study had advanced NVG, which may be one of the reasons why anti-VEGF treatment showed no significant therapeutic effect. Therefore, further studies are needed to determine whether anti-VEGF therapy shows a positive effect on the surgical outcome of NVG, particularly during the early stages. In addition, there is often a selection bias in patients receiving anti-VEGF drug therapy at present. In general, patients with more severe diseases are recommended to receive anti-VEGF drug therapy.

Different opinions concerning whether primary diseases in patients will affect surgical effects are noted in the present study. Every et al discovered in their research that the surgical success rate between CRVO patients receiving drainage valve implantation and those without CRVO was comparable 30. Nevertheless, the success rate is related to the progress of primary vascular diseases. Mermoud et al suggested that surgical effects in diabetics were superior to those in central retinal vein occlusion (CRVO) patients 21. Hayreh considered that patients with CRVO as the primary disease generally had more severe illness than diabetics and NVG patients, thus, affecting the surgical outcome and prognosis 4. In the study of Ye H, the difference in the surgical success rates among CRVO patients, diabetics, and patients with other diseases was not statistically significant 31. Similarly, our results show no statistical differences related to other diseases in our study. However, we cannot make definitive conclusions due to sample size.

Finally, the absence of postoperative complications was an influencing factor for the success rate at 1 year (P<0.05). Postoperative complications after AGV may include postoperative anterior chamber hyphema, shallow anterior chamber, malignant glaucoma, choroidal detachment, corneal endothelial decompensation, drainage valve exposure, and drainage valve displacement. In our study, the shallow AC rate was 9%, due to over aqueous humor filtration. Our research showed that postoperative low-tension shallow anterior chamber overwhelmingly belonged to grade I according to the 3-grade classification, which could be cured with conservative treatment. Although it was the second most common complication after AGV in our study, we cannot conclude that the occurrence of shallow AC was related to surgical success rate.

In summary, AGV implantation is effective and safe for treatment of NVG. Age, standardized retinal photocoagulation history, no postoperative complications and postoperative anterior chamber hyphema are influencing risk factors for the success of AGV surgery. Careful operation should be performed during the operation, complications should be avoided, and standardized retinal photocoagulation should be performed in time after the operation.

## Figures and Tables

**Table 1:** Basic Data of Patients Enrolled

Characteristics	
**Gender**	
Male	49 (74.2%)
Female	17 (25.8%)
Age (Year)	50.68 ± 13.73
**Eye**	
Left-sided	32 (48.5%)
Right-sided	34 (51.5%)
Mean Course of disease (Month)	9.27 ± 14.43
Mean Pre- BCVA	2.01 ± 1.34
Mean Pre- IOP (mmHg)	39.30 ± 12.19
Mean Pre-medications No.	2.58 ± 0.56
Mean 1-year post-medication No.	1.03 ± 1.05
PRP history	40 (60.6%)
Pre-anti-VEGF history	31 (47.0%)
**Lens status**	
Phakic	44 (66.7%)
Pseudophakic	18 (27.3%)
Aphakic	4 (6.1%)
**Primary Diseases**	
CRVO+BRVO	7 (10.6%)
DM	41 (62.1%)
PACG	4 (6.1%)
Trauma	5 (7.6%)
**Previous History**	
PPV	8 (12.1%)
Cataract surgery	13 (19.7%)
Trabeculectomy	1 (1.5%)
Transscleral cyclophotoculation	2 (3.0%)

CRVO: central retinal vein occlusion, BRVO: Branch retinal vein occlusion, DM: Diabetes mellitus, PACG: Primary angle-closure glaucoma, PPV: Pars plana vitrectomy

**Table 2:** Post-operative Complication Rates

Complication	N (%)
Hyphemia	10 (15.2%)
Shallow anterior chamber	9 (13.6%)
Choroidal detachment	1 (1.5%)
Corneal decompensation	1 (1.5%)
Hypotony maculopathy	1 (1.5%)
Drainage tube exposure	1 (1.5%)
Drainage tube displacement	1 (1.5%)
Malignant glaucoma	2 (3%)

**Table 3:** Stepwise Regression Analysis of Factors Influencing Surgical Success Rate at 1 Year after Surgery

Factors	B	S.E.	Wald	DOF	P	Exp (B)
Age	-0.083	0.031	7.296	1	0.007	0.92
No post-operative complication			9.443	3	0.024	
Hyphema	4.622	1.524	9.205	1	0.002	101.735
Shallow anterior chamber	0.631	1.151	0.301	1	0.583	1.88
Other	0.304	0.874	0.121	1	0.728	1.355
PRP history	2.574	0.858	8.996	1	0.003	13.117

B, the slope of the line; S.E., standard error; Wald, Wald test; DOF, degree of freedom; P, p-value; Exp (B), odds ratio.
